# Diet of Free Ranging American Mink (*Neovison vison*) in Denmark

**DOI:** 10.3390/ani13030461

**Published:** 2023-01-28

**Authors:** René Worup Rørbæk, Tobias Astell Andersen, Cino Pertoldi, Alex Jørgensen, Sussie Pagh

**Affiliations:** 1 Department of Chemistry and Bioscience—Section of Biology and Environmental Science, Aalborg University, Fredrik Bajers Vej 7, 9220 Aalborg, Denmark; 2 Aalborg Zoo, Mølleparkvej 63, 9000 Aalborg, Denmark

**Keywords:** invasive species, diet analysis, stomach contents, captive-born free-ranging mink, diet composition, Danish mink population

## Abstract

**Simple Summary:**

This study is the first to compare the diets of captive-born (escaped farm mink) and wild-born mink. This study aimed to evaluate the diets of captive-born and wild-born mink and compare the diets of mink on mainland Denmark and the seasonal variations in diet. The stomachs of 243 wild-born and 114 captive-born mink were analyzed. No significant differences were found between the diets of captive-born mink and wild-born mink from the two populations (mainland Denmark and Bornholm). Significantly more empty stomachs were found during spring than during the summer and autumn. Primarily, the diets of the mink consisted of fish, voles, birds, and crustaceans.

**Abstract:**

Non-native American mink (*Neovison vison*) is a widely spread predator in Denmark. The feral population in mainland Denmark consists of captive-born mink that escaped from fur farms and wild mink born in nature, whereas the population on Bornholm is almost all wild-born mink. In this study, the diets of feral mink in mainland Denmark and on the island of Bornholm are analyzed. The aim of this study was to examine (1) whether the diet of the larger captive-born mink differs from that of the smaller wild-born mink, (2) assess the regional variations between the diets of mink in mainland Denmark and on Bornholm, and (3) investigate the seasonal variation in the diet composition of mink. The stomach contents of 364 mink (243 wild-born and 114 captive-born) culled in the years 2019–2022 were analyzed. Of these, 203 mink were from mainland Denmark, and 154 were from Bornholm. No significant differences were found between the diets of captive-born mink and wild-born mink or the mink found in mainland Denmark and on the island of Bornholm. Significantly more empty stomachs were found during spring than during the summer and autumn, suggesting a bottleneck in the diet during spring.

## 1. Introduction

The American mink (*Neovison vison*), hereafter referred to as mink, is a non-native species in Denmark. Originally, the mink was brought to Denmark during the establishment of fur production in the 1920–1930s [1]. Minks are semi-aquatic mammals belonging to the Mustelidae family. They can live near both freshwater and saltwater habitats. The population of mink in Denmark was relatively low until the 1980s, whereafter the Danish gamebag statistics of mink increased from around 1000 to 8000 minks near the millennium. In 2021/22, the Danish gamebag statistics for mink was 768 individuals [2]. Although the Danish feral mink population has decreased in recent decades, minks are still widely distributed across the country [2,3].

A previous study of freeranging mink in Denmark showed that the feral mink population consists of two subpopulations of mink: wild-born and captive-born mink [4]. In previous Danish studies, 80% of the feral mink caught by hunters in the years 1998–2000 and approximately 30% of the mink caught during the winters of 2014–2018 were found to be mink born on farms [4,5]. Former diet studies of Danish mink have never examined the captive-born mink diet and tested whether there are differences in the diet of the two sub-populations.

Minks have a broad diet depending on habitat and season [6,7]. However, in most habitats, the general diet consists of small mammals, fish, amphibians, birds, and invertebrates [7,8,9]. A previous Danish study showed that the main diet of mink consisted of small mammals and invertebrates, birds, fish, and amphibians [8]. In agricultural parts of Poland, mammals were likewise the most common prey (43%), and birds were less common (8%), whereas in the wetlands of Poland, birds (60%) were more frequently found than mammals (51%), fish (22%), and amphibians (8%) [10,11].

In the wetlands of Estonia, amphibians (65%) and mammals (30%) were found to be the most common prey [12]. In Sweden, fish (36%) and mammals (33%) made up the bulk of the diet of mink [13]. Additionally, most of the studies found that the diet composition of mink changed during seasons, showing the opportunistic hunting strategy of going after the easiest target available under the current conditions [10,11,13].

Especially in wetlands and on islands, mink may have an impact on the breeding success of ground-nesting birds, and in some areas, mink may also influence populations of the European water vole (*Arvicola amphibius)* [11,14,15,16,17]. The habitat and the body size of the mink may influence the choice of prey. In Scotland, the larger males preyed more upon lagomorphs, while females preyed more upon fish and crustaceans. These differences were consistent in each season, except the autumn, wherein the males preyed more heavily upon fish and crustacea than females [18].

From 2007 to 2018, the number of farmed male mink pelts longer than 101 cm increased by 30%. Likewise, the number of female mink pelts longer than 83 cm increased by 24%, and moreover, both breeding males and females have increased their mean weight by 70% for the past 10–15 years [4].

Larger body size may not only influence the size of mink prey, but may also have a negative influence on survival under natural conditions. In a survey of the body size of mink during their colonization of Warta Mouth National Park, west Poland, the body size of mink changed significantly from 1996 to 2004 [19]. The mean body weight of males dropped by 13% from 1.36 to 1.18 kg, and that of females dropped by 16% from 0.83 to 0.70 kg [19]. These changes were ascribed to changes in food availability [19]. Natural selection pressure is relaxed in captive-born mink [20]. Therefore, the effect of natural selection on captive-born mink is expected to be strong immediately after escaping from a farm. Generations of mink living in the wild must adapt their body size, color, behavior, and biology to be able to survive under natural conditions [19]. The minks that have escaped from Danish farms have previously shown a 75% risk of dying during the first three months in the wild [21]. In contrast, wild-born minks have a 70% risk of dying during their first year [22]. It is therefore expected that mink that have been raised on farms will be less adapted to catch live prey and that the prey of newly escaped mink may differ from mink born in nature.

The aims of this study were to investigate the following:

(1) Whether the diet of the larger captive-born mink differs from that of the smaller wild-born mink.
(2) If there are regional variations between mink diet on mainland Denmark and Bornholm.
(3) If there are seasonal variations in the diet composition of mink in Denmark.

## 2. Materials and Methods

### 2.1. Study Area

**Figure 1:**
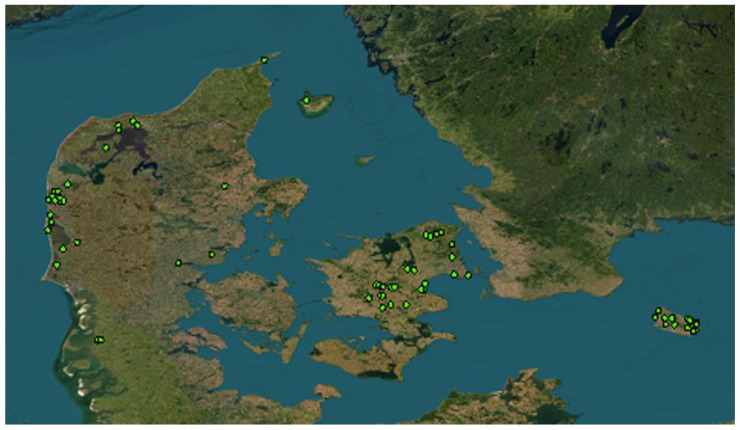
A map of Denmark showing Jutland, Zealand, and Bornholm as well as the geographical distribution of mink (green dots) caught by hunters during this study.

The minks were collected in Denmark during 2019–2022 and divided into two groups: mainland Denmark (Jutland and Zealand) and the island of Bornholm. The Danish peninsula Jutland covers an area of 29.775 km^2^ and is bordered by Germany to the south. The northernmost point of Jutland is located at 57°43′ N/10°37′ E. Zealand covers 7.031 km^2^ and Bornholm 588.36 km^2^ (Figure 1).

Denmark is characterized by flat arable land and sandy coasts, low elevation, and a mild coastal temperate climate. Around 70% of the country comprises intensively human-modified agricultural land. Bornholm differs geologically and in natural conditions from the rest of Denmark by having rockier ground similar to Swedish nature and having no native predators [4].

Though the two largest Danish regions (Jutland and Zealand) are isolated by the Great Belt, the mink populations live under similar natural conditions. The natural habitats, arable lands in the two regions and the native predators (e.g., red fox, *Vulpes vulpes*; badger; *Meles meles*, otter; *Lutra lutra*, and other mustelids), are comparable. Additionally, the proportion of captive-born mink is similar, around 30% [4]. Bornholm is located in the Baltic Sea (around 200 km) from mainland Denmark. The minks on Bornholm are considered a true feral population since only 1% of escaped captive-born mink were found, and this is due to the fact that the island is isolated [4].

### 2.2. Collection of Material

Most of the minks were trapped in kill traps or live-catch traps by hunters. Few of the minks were shot during hunts or roadkill. The hunters submitted information relating to each mink, including the date and location of when the mink was killed. The minks were delivered to the University of Aalborg or The Technical University, Lyngby. The minks were kept at −20 °C until the necropsy. During the necropsy, the minks were sexed, measured from nose to tail and tail to the tip of the last vertebra, and the color of the fur was recorded. The gastrointestinal tracts, sexual organs, and mandible were removed and stored at −20 °C for further examination.

A recent study has shown that Danish captive-born mink can be separated from wild-born mink by body length [22]. The wild-born mink and captive-born mink can be separated when they are older than four months. Hence, the mink in this study were divided into wild-born and captive-born mink accordingly [22]. In this study, minks with a body length below 43 cm and 39 cm for males and females, respectively, were considered to be wild-born mink.

### 2.3. Diet Analysis of Mink

The gastrointestinal tracts of the mink were stored at −20 °C before the analysis. Only the stomach contents were used for the analysis due to prior studies finding no significant difference between the contents of the intestines and the stomachs in mink [8].

The analysis was performed using the same procedure as described by [23,24]. The weight of the stomachs was noted before and after the stomachs were emptied to determine the total mass of the stomach contents. The prey categories’ volumes were estimated to the nearest five percent. The mammals were identified by unique hair characteristics, following [25,26]. Bird remains were identified by the shape of the nodes on the individual feather barbules under a microscope [24,27].

Fish were identified by their vertebrae and scales [28,29]. Crustaceans and insects were not identified to a lower taxon, and plant material was considered to be ingested unintentionally along with prey.

Mink hairs were found in most stomachs, but were considered to be ingested during grooming and were, therefore, not included in the results.

The data were grouped based on sex, season, region, and origin of the mink. For each prey category, the percent frequency of occurrence (%Occ) and percent biomass (%Bio) were calculated:

(1) $$\%Occ=\frac{n_{Prey}}{n_{Total\;per\;group}}$$

$n_{Prey}$ is the total number of preys found in the mink, and $n_{Total\;per\;group}$ is the total number of mink in the different groups.

The biomass was calculated using the volume of prey categories $\left(V_{Prey}\right)$ times the weight of the stomach contents ($Weight_{Stomach}$):

(2) $$Biomass=V_{Prey}\;\cdot \;Weight_{Stomach}$$

Additionally, percent biomass (%Bio) was the biomass per prey category (Biomass) divided by the sum of the total biomass per group ($Biomass_{Total})$.

(3) $$\%Bio=\frac{Biomass}{Biomass_{Total}}$$

### 2.4. Statistical Analysis

G-tests were used to test if there were any differences between the number of empty stomachs between wild-born and captive-born mink, likewise across regions and seasons. The nonparametric multivariate test, one-way PERMANOVA (permutational MANOVA or NPMANOVA), was used to test for the differences between the prey biomass in captive-born and wild-born mink, as well as the regional and seasonal variation in the biomass of the prey in the wild-born mink [30,31]. The nonparametric test, PERMANOVA, was chosen due to the data not following a normal distribution (Shapiro–Wilk’s W: 0.25, *p* = 5.55 × 10^−33^). All of the statistical tests were performed using the software of R version 4.0.3 [32].

## 3. Results

### 3.1. Empty Stomachs

Of the 364 analyzed stomachs, 163 (44.7%) were empty (Table 1). No significant differences were found in the number of empty stomachs between the wild-born (43%) and captive-born mink (50%) (G-test: G = 0.23, *p* > 0.05) (Table 1). Likewise, no significant differences in the number of empty stomachs were found between the minks from mainland Denmark (46%) and the minks from Bornholm (46%) (G = 0.15, *p* > 0.05) (Table 1). However, there was a significant difference in the number of empty stomachs between seasons: spring (62%), summer (44%), autumn (40%), and winter (52%), respectively (G = 7.64, *p* = 0.032) (Table 1). Additionally, the pairwise G-test showed that spring was significantly different from summer and autumn (Table A1).

### 3.2. Prey of Wild-Born Mink

Fish were the most frequently occurring prey found in the stomachs of wild-born minks (29%), the most predominant prey being carp fish (*Cyprinidae*) (15%) (Table 2). Birds were the second most frequent prey group, found in 26% of the stomachs, with the most predominant bird being landfowl (*Galliformes*), which was found in 11% of the wild-born mink (Table A2). The third most frequent prey group was small mammals, which occurred in 21% of the stomachs. The most common mammal preys were voles (*Cricetidae*) (10%) (Table 2).

The prey category with the highest biomass found in wild-born mink was carp fish (12%), followed by voles (11%), landfowl (7%), and crustaceans (*Crustacea*) (6%) (Table 2).

### 3.3. Prey of Captive-Born Mink

Mammals and birds were the most frequent prey groups found in the stomachs of the captive-born mink; both groups were found in 29% of the stomachs. The most common mammals were voles and mice (*Muridae)*, which were found in 12% of the stomachs, followed by landfowl and waterfowl (*Anseriformes)*, found in 9% of the stomachs (Table 2). Fish were the third most frequent prey type, found in 20% of the stomachs (Figure 1). Carp fish were found in 12% of the stomachs (Table 2).

The prey category with the highest biomass found in captive-born mink was birds, representing almost one-third (30%) of the biomass, with waterfowls constituting 13%, and gulls (*Larinae)* (9%).

Crustaceans, insects (*Insecta*), amphibians (*Amphibia*) and reptiles (*Reptilia*) were found less frequently—10%, 8%, 4%, and 2%, in wild-born mink, and 6%, 8%, 3%, and 0% in captive-born mink. The lesser prey categories were more often found in wild-born mink, but not significantly (Table 2).

### 3.4. No Significant Differences between the Diet of Wild-Born and Captive-Born Mink

No significant differences were found in the biomass of prey between wild-born and captive-born mink with either MANOVA (Wilks Lambda: 0.98, F = 1.4, *p* = 0.25) or PERMANOVA tests (F = 2.3, Permutation N = 9999, *p* = 0.07).

### 3.5. Regional Trends in the Composition of Mink Diet

In mainland Denmark, the most frequent prey group eaten by mink was birds, found in 37% of the mainland mink, with the most common bird prey being landfowl (14%) (Table 3). Fish was the second most common prey group, found in 22% of the stomachs of mainland mink. The most predominant fish were carp fish, appearing in 13% of the mainland minks. The least common main prey group was mammals, which made up 21% of the stomachs, with the main mammals being voles (10%) (Table 3).

The prey group with the highest biomass found in mainland mink was birds, representing 26% of the total biomass, with both waterfowl and landfowl representing approximately 9% of the total biomass found in mainland mink stomachs. Carp fish and voles represented 8% and 6% of the total mainland biomass, respectively.

The most common prey group eaten by mink on Bornholm was fish, being found in 25% of the stomachs on Bornholm (Table 3). The most predominant fish was carp fish, found in 15% of the mink on Bornholm (Table 3). Mammals were the second most common prey group found in mink on Bornholm (22%), with voles being the predominant mammal, which was found in 11% of the stomachs. Birds were the least common prey group found on Bornholm, being present in 12% of the mink (Table 3).

The prey category with the highest biomass found in mink on Bornholm was mammals, representing 23% of the total biomass found in mink on Bornholm. However, the individual prey category with the highest biomass was carp fish, representing 15%, while voles represented 12% of the total biomass. Therefore, even though carp fish had the largest biomass found in the mink diet on Bornholm, mammals (shrews (*Sorex*), mice and voles) still had a higher proportion of the total biomass than fish (perch (*Percidae*), carp and salmon (*Salmonidae*)), when looking at all prey categories (Table 3).

### 3.6. No Significant Differences in the Diet of Mink between Mainland Denmark and Bornholm

No significant differences were found in the biomass of prey between the mink from Bornholm or the mink from mainland Denmark when using either MANOVA (Wilks Lambda: 0.98, F = 0.78 *p* = 0.54) or PERMANOVA tests (F = 0.59, Permutation N = 9999, *p* = 0.64).

### 3.7. No Seasonal Significant Difference in Prey Selection

Birds were the most frequent prey for mink during all seasons except for autumn, when mammals had a frequency of 29% (Table 4). The second most frequent prey was fish during spring (25%) and autumn (26%) and mammals during winter (17%), while fish (24%) and mammals (24%) were tied during the summer (Table 4). The most common prey categories found during the seasons were landfowl during spring (17%) and winter (21%), carp fish during the summer (15%), and mice during the autumn (16%) (Table 4).

The prey with the highest percent biomass found in the stomachs during the seasons were waterfowl in spring (30%), voles during the summer (16%) and autumn (23%), and crustaceans during winter (19%), showing that even though birds such as landfowl were the most common prey category found in the stomachs during the spring and winter, respectively, they were not the prey category representing the highest amount of the biomass found during the seasons (Table 4).

No significant seasonal variations were found in the biomass of prey in Danish mink when using either MANOVA (Wilks Lambda: 0.97, F = 0.82, *p* = 0.64) or PERMANOVA tests (F = 0.82, Permutation N = 9999, *p* = 0.61).

## 4. Discussion

### 4.1. Wild-Born and Captive-Born Stomach Contents and Differences

A significant proportion of the stomachs of both wild-born and captive-born mink were empty when necropsied, indicating that mink enter the traps when hungry. The Danish Nature Agency recommends that traps are baited with canned fish, e.g., marine species or cat pellets [33]. However, it cannot be excluded that hunters may have used other easily available bait, e.g., landfowl, that may be confused with wild caught prey.

The farmed minks were expected to be poorly adapted to natural conditions, only having a few months to acclimatize, a lower fitness due to their large body size, a lack of practice with live prey, and a fur color that may not provide camouflage [20,21,34,35]. If captive-born minks are not able to acclimatize, they are not expected to survive in the wild. However, in this study, no significant differences were found between the stomach contents of the wild-born and captive-born mink. Additionally, a similar amount and variety of prey were found in the wild-born and captive-born mink, pointing to the fact that captive-born mink are able to catch prey in the wild. The absence of maggots or carrion beetles indicated that neither the wild-born nor captive-born mink were feeding on decaying carrion. A higher mortality rate would be expected if the captive-born mink were not able to find food in the wild. However, the mortality rates found in Danish wild-born and captive-born free-ranging mink indicate that farmed mink may survive more than a few months [4,5].

However, the stomachs of wild-born mink tended to contain a higher biomass of voles and crustaceans than those of the captive-born. These are prey types that minks are expected to catch under natural conditions [9,10,36,37]. On the contrary, the stomachs of captive-born mink tended to contain more birds, especially waterfowl and gulls. Captive-born mink can be found in harbors around Denmark and may be more adapted to human activities.

### 4.2. Competition with Other Predators

During the past two decades, the European otter has become more abundant in mainland Denmark, especially in Jutland [38]. Otters might compete with the mink and, therefore, force the mink to hunt more terrestrial prey [39,40]. However, this disagrees with a newer study, which found that mink and otters in Poland could coexist in aquatic habitats [41]. The diet of mink from Bornholm may be more easily accessible due to the absence of red foxes and otters on Bornholm, which allows mink to predate on small rodents and fish. A Swedish study found that the absence of the red fox in Sweden led to a growth in the mink population and suggested that the increase in the mink population was due to access to new terrestrial prey because of the lack of competition with foxes [42].

### 4.3. Seasonal Variation in the Diet of Mink

The basic mink diet in all seasons consisted of fish, mammals and birds, and no clear seasonal differences were found in the main prey in Denmark during the seasons. This agrees with other studies relating to mink diets [8,10,11,13].

Fish were most abundant during summer and autumn, especially carp fish. The increase in carp fish in the diet during the summer and autumn could be due to lower water levels, overgrowth, and the partial drying out of rivers, leading fish to crowd in shallow waters, making them easier targets for mink [10]. Mammals were most frequent during autumn, which is the same tendency that was observed in a previous Danish study of mink diets, considering that it was due to the higher population size of smaller mammals during autumn [8].

## 5. Conclusions

The study shows that captive-born mink can survive and catch live prey. Having been raised on a farm, captive-born mink were expected to be less well adapted to life in the wild. However, no differences were found in diet between wild-born and captive-born mink neither in occurring of species nor in amount of stomach content, indicating that they are capable of hunting prey to the same standard as wild-born mink. No regional variations were found between the mink from Bornholm and the mink from mainland Denmark. The main diet of the mink in both regions was composed of fish, mammals and birds. A significant higher number of empty stomachs during spring may mean that, especially the larger captive-born mink, experiences a bottleneck in diet during this time of year. This is probably the driving force in the selection of smaller body size in feral mink.

## Figures and Tables

**Table 1:** Number of stomachs and percentage of empty stomachs in relation to wild-born, captive-born, regions (mainland Denmark and Bornholm), and seasons (spring, summer, autumn and winter), the weight of the stomachs with content.

Mink	Stomachs (*n*)	Stomachs with Content (*n*)	Weight (g) (CI95%)	Percentage Empty Stomachs (%)
Wild-born	248	136	12.7 (2.3)	45.2
Captive-born	124	64	15.3 (3.8)	48.4
Mainland Denmark	211	115	14.2 (2.9)	45.5
Bornholm	158	85	12.5 (2.3)	46.2
Spring	89	34	13.1 (2.4)	61.8
Summer	89	50	12.5 (3.6)	43.8
Autumn	92	55	13.5 (3.4)	40.2
Winter	63	30	15.1 (6.5)	52.4

**Table 2:** Occurrence (Occ), percentages of occurrence (%Occ), and percentage of biomass (%Bio) for wild-born mink (*n* = 136) and captive-born mink (*n* = 65).

	Wild-Born (*n* = 136)			Captive-Born (*n* = 65)		
Prey Group	Occ	%Occ	%Bio	Occ	%Occ	%Bio
**Mammals**	29	21.3	17.2	19	29.2	10.7
*Sorex*	3	2.2	0.2	2	3.1	0.5
*Muridae*	11	8.1	5.0	8	12.3	7.3
*Cricetidae*	13	9.6	10.8	8	12.3	2.9
**Birds**	35	25.7	12.6	19	29.2	29.6
*Galliformes*	16	11.8	6.7	6	9.2	6.2
*Passeriformes*	11	8.1	3.3	3	4.6	1.4
*Larinae*	3	2.2	0.5	4	6.2	9.1
*Anseriformes*	5	3.7	2.2	6	9.2	12.8
**Fish**	40	29.4	21.3	13	20.0	8.3
*Percidae*	8	5.9	4.7	4	6.2	1.6
*Cyprinidae*	20	14.7	12.4	8	12.3	5.4
*Salmonidae*	6	4.4	2.5	0	0	0
*Anguilliformes*	1	0.7	0.1	0	0	0
*Gasterosteidae*	1	0.7	0.4	0	0	0.0
**Others** *Crustacea*	13	9.6	6.1	4	6.2	0.6
*Insecta*	11	8.1	0.8	5	7.7	0.7
*Amfibia*	5	3.7	1.7	2	3.1	4.8
*Reptilia*	2	1.5	0.5	0	0	0
*Plantae*	36	26.5	3.5	24	36.9	4.3

**Table 3:** Occurrence (Occ), percentages of occurrence (%Occ), and percentage of biomass (%Bio) for mink in mainland Denmark (*n* = 111) and mink on Bornholm (*n* = 83).

Region	Mainland Denmark (*n* = 111)			Bornholm (*n* = 83)		
	Occ	%Occ	%Bio	Occ	%Occ	%Bio
Prey Groups						
**Mammals**	23	20.7	9.2	18	21.7	22.8
*Sorex*	4	3.6	0.6	1	1.2	0.2
*Muridae*	8	7.2	3.0	8	9.6	10.5
*Cricetidae*	11	9.9	5.6	9	10.8	12.0
**Birds**	41	36.9	25.5	10	12.0	7.4
*Galliformes*	16	14.4	9.2	6	7.2	1.2
*Passeriformes*	8	7.2	1.7	3	3.6	4.2
*Larinae*	7	6.3	5.3	0	0.0	0.0
*Anseriformes*	10	9.0	9.3	1	1.2	2.0
**Fish**	24	21.6	13.3	21	25.3	21.0
*Percidae*	8	7.2	4.6	3	3.6	1.7
*Cyprinidae*	14	12.6	8.2	12	14.5	14.9
*Salmonidae*	0	0.0	0.0	6	7.2	4.5
*Anguilliformes*	1	0.9	0.1	0	0.0	0.0
*Gasterosteidae*	1	0.9	0.4	0	0.0	0.0
**Others** *Crustacea*	7	6.3	4.1	10	12.0	4.2
*Insecta*	7	6.3	0.5	9	10.8	1.4
*Amphibia*	2	1.8	2.6	5	6.0	3.1
*Reptilia*	1	0.9	0.4	1	1.2	0.2
*Plantea*	34	30.6	3.4	21	25.3	4.4

**Table 4:** Occurrence (Occ), percentages of occurrence (%Occ), and percentages of biomass (%Bio) for mink (*n* = 166) and mink during the seasons: spring (*n* = 36), summer (*n* = 46), autumn (*n* = 55) and winter (*n* = 29).

Seasons	Spring (*n* = 36)			Summer (*n* = 46)			Autumn (*n* = 55)			Winter (*n* = 29)		
	Occ	%Occ	%Bio	Occ	%Occ	%Bio	Occ	%Occ	%Bio	Occ	%Occ	%Bio
Prey Groups												
**Mammals**	7	19.4	14.1	11	23.9	15.3	16	29.1	18.8	5	17.2	5.4
*Sorex*	0	0	0.0	2	4.3	0.5	1	1.8	0.1	2	6.9	2.0
*Muridae*	4	11.1	10.1	4	8.7	4.7	6	10.9	3.7	0	0	0.0
*Cricetidae*	3	8.3	4.0	5	10.9	10.1	9	16.4	14.9	3	10.3	3.4
**Birds**	13	36.1	30.6	12	26.1	15.7	12	21.8	16.6	11	37.9	26.4
*Galliformes*	6	16.7	9.8	4	8.7	6.9	4	7.3	3.6	6	20.7	8.0
*Passeriformes*	2	5.6	0.7	4	8.7	1.4	3	5.5	6.4	1	3.4	2.6
*Charadriiformes*	2	5.6	2.3	2	4.3	5.6	1	1.8	0.2	2	6.9	10.0
*Anseriformes*	3	8.3	17.9	2	4.3	1.8	4	7.3	6.4	2	6.9	5.9
**Fish**	9	25.0	9.3	11	23.9	22.4	14	25.5	17.5	2	6.9	10.2
*Percidae*	2	5.6	0.9	2	4.3	8.8	5	9.1	4.0	0	0.0	0.0
*Cyprinidae*	5	13.9	6.7	7	15.2	9.8	6	10.9	11.2	2	6.9	10.2
*Salmonidae*	2	5.6	1.8	2	4.3	3.7	1	1.8	1.0	0	0	0.0
*Anguilliformes*	0	0	0.0	0	0	0.0	1	1.8	0.1	0	0	0.0
*Gasterosteidae*	0	0	0.0	0	0	0.0	1	1.8	1.1	0	0	0.0
**Others** *Crustacea*	4	11.1	4.1	2	4.3	0.6	5	9.1	1.6	3	10.3	12.0
*Insecta*	3	8.3	0.4	5	10.9	0.9	3	5.5	0.5	1	3.4	0.4
*Amfibia*	0	0	0.0	3	6.5	3.9	1	1.8	1.5	0	0	0.0
*Reptilia*	0	0	0.0	1	2.2	0.2	1	1.8	1.1	0	0	0.0
*Plantae*	5	13.9	1.0	10	21.7	1.0	21	38.2	4.8	13	44.8	1.4

## Data Availability

The data presented in this study are available upon request from the corresponding author.

## Appendix A

**Table A1:** G-tests’ g-values and *p*-values in parentheses between the number of empty stomachs and the different seasons. Significant *p*-values are tagged with an asterisk.

Season G-Test	Spring (*p*-Value)	Summer (*p*-Value)	Autumn (*p*-Value)	Winter (*p*-Value)
Spring		5.1 (0.02) *	7.6 (0.006) *	2.5 (0.11)
Summer	5.1 (0.02) *		0.12 (0.73)	0.01 (0.77)
Autumn	7.6 (0.006) *	0.12 (0.73)		0.55 (0.46)
Winter	2.5 (0.11)	0.01 (0.77)	0.55 (0.46)	

**Table A2:** The mean biomass with standard deviation (±STD) and the 95% confidence intervals (CI95) for the main diet prey categories. The table compares the diet of wild-born and captive-born mink, diet of mink on Bornholm with that of mainland Denmark, and the diet of mink between seasons.

	Prey	Biomass	STD	CI95
Wild-born	Mammals	15.3	17.8	7
	Fish	14.5	14.4	5.2
	Birds	9.4	12	4.1
	Crustaceans	11.6	15.3	8.3
	Insects	1.9	1.2	0.7
Captive-born	Mammals	9.4	11.8	5.9
	Fish	11.7	18.4	11.5
	Birds	23.1	25.9	12.3
	Crustaceans	2.1	1.3	1.3
	Insects	2	1.9	1.7
Bornholm	Mammals	17.2	18	8.3
	Fish	13.6	12.1	5.2
	Birds	10.1	15.4	9.6
	Crustaceans	5.7	5.7	3.6
	Insects	2.1	1.1	0.7
Mainland Denmark	Mammals	9.9	13.1	5.2
	Fish	13.7	16.7	6.7
	Birds	15.5	19.8	6.1
	Crustaceans	14.6	19.5	14.4
	Insects	1.7	1.8	1.3
Spring	Mammals	16.3	14.8	11
	Fish	8.4	8.3	5.4
	Birds	19	25.8	14
	Crustaceans	8.3	8.3	8.1
	Insects	2	0.6	0.7
Summer	Mammals	11.9	19.4	11.5
	Fish	17.4	16.7	9.9
	Birds	11.2	14.8	8.4
	Crustaceans	2.6	2.6	2.2
	Insects	1.6	1.6	1.4
Autumn	Mammals	11.6	15.6	7.6
	Fish	12.3	13.4	7
	Birds	13.6	15.1	8.5
	Crustaceans	3.1	1.6	1.4
	Insects	1.5	0.7	0.8
Winter	Mammals	6.4	3.9	3
	Fish	30.3	29.1	29.3
	Birds	8	12.1	7.7
	Crustaceans	23.8	23.6	22.7
	Insects	2.2	1.8	1.5
